# The effect of workload on primary care doctors on referral rates and prescription patterns: evidence from English NHS

**DOI:** 10.1007/s10198-024-01742-7

**Published:** 2024-12-07

**Authors:** Hanifa Pilvar, Toby Watt

**Affiliations:** 1 University of Oxford, Oxford, UK; 2 The Health Foundation, London, UK

**Keywords:** Primary care workforce, Physicians’ workload, Referral decisions, Prescription patterns, Antibiotics, General practitioner, I10, I11, J81

## Abstract

This paper investigates the impact of workload pressure on primary care outcomes using a unique dataset from English general practices. Leveraging the absence of General Practitioner (GP) colleagues as an instrumental variable, we find that increased workload leads to an increase in prescription rates of antibiotics as well as in the share of assessment referrals. On the other hand, the quantity and frequency of psychotropics decreases. When there is an absence, workload is intensified mostly on GP partners, and the mode of consultation shifts toward remote interactions as a response to higher workload pressure. The effects are more pronounced for patients above 65 years-old and those in Short-staffed practices. Our study sheds light on the intricate relationship between workload pressure and patient care decisions in primary care settings.

## Introduction

In this paper, our objective is to investigate the implications of greater physician work- load on outcomes in primary care. Over time, there has been a consistent rise in workload pressures faced by General Practitioners (GPs) due to the increasing levels of illness and ag-Ing population and inadequate supply of GPs [12, 17]. This trend raises significant concerns regarding the potential impact of under-staffing on the quality and consistency of primary care delivery. Such an intensified workload has the potential to influence and distort the decision-making process of GPs. Consequently, our primary goal is to causally identify and analyse the relationship between GP workload, measured by the daily number of appointments per GP, and its influence on referral and prescription frequencies.

**Fig. 1:**
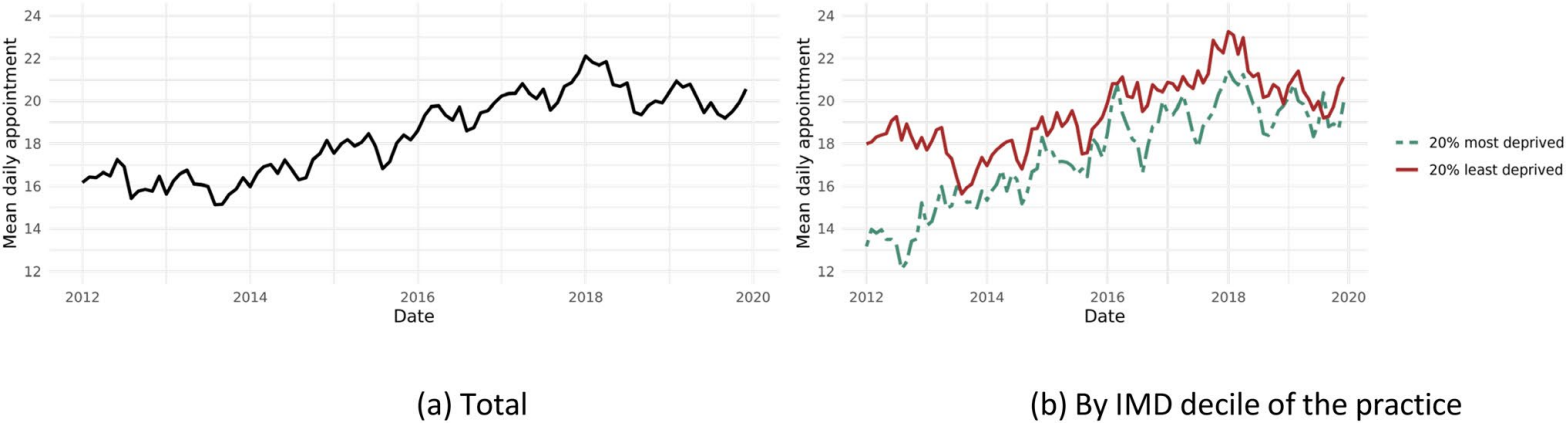
Average number of appointments per day per GP

The workload pressure in English primary care has experienced a significant and rapid increase in recent years. As depicted on the left side of Fig. 1, we observe a continuous upward trend in the average number of appointments per GP per day from 2012 to 2019. Over a span of merely eight years, GPs have been tasked with an additional four appointments per day on average.

In addition to the overall increase in daily GP appointments over time, there is notable cross-sectional variation in GP workload. For instance, GPs working in more deprived practices experience significantly higher workloads, as shown in the right panel of Fig. 1. This exacerbates inequality levels, as GPs in deprived areas often face more complex cases with higher rates of long-term illness and frequent consultations for both acute and chronic conditions [29]. The average daily appointments per GP range from 16 to 39 at the practice level, indicating significant disparities in workload across practices (see Fig. 6).

For our research, we utilize an extensive dataset comprising pseudonymized patient- level records obtained from the Clinical Practice Research Datalink (CPRD). Our analysis focuses on a sample of 50 primary care practices located in London, serving a population of approximately 600,000 patients from 2012 to 2019.

Identifying the impact of workload on patient outcomes is not straightforward due to the endogenous linkage between workload, staffing decisions, and doctors’ sorting into practices. These endogenous decisions remain unobserved in the data. Consequently, we require an exogenous variation in workload to establish the causal link between workload and patients’ outcomes. We employ the short-term absence of a GP colleague as an instrumental variable (IV) for the number of daily appointments per GP. Our first-stage results reveal that the absence of one GP increases the number of appointments for present GPs by 0.36. We demonstrate that the majority of this workload pressure is on partner GPs who are practice owners. Temporary GPs account for a smaller fraction of the additional workload pressure while the pressure on salaried GPs and nurses does not change.

Measuring primary care outcomes presents challenges; therefore, our analysis narrows down to two pivotal decisions made by GPs: referrals to secondary care and prescription frequencies and volumes. We also look at particular categories of prescriptions such as frequent drugs, opioids, antibiotics, Quality and Outcome Framework (QOF) incentivised drugs and mental health drugs. These outcomes hold significant importance in primary care, particularly in England, where GPs serve as the gatekeepers of the entire healthcare system. On one hand, referrals to secondary care enable the appropriate allocation of patient needs to specialized hospital services. On the other hand, primary care plays a crucial role in managing both chronic and acute illnesses, alleviating the burden on other healthcare sectors. Effective management of illnesses relies, in part, on employing sound prescribing practices.

Two potential hypotheses can be proposed to explain the relationship between workload pressure and the outcomes of primary care appointments. The first hypothesis suggests that doctors, when under pressure, may opt for more intensive and conservative decisions because they have less time to investigate patients’ symptoms. This is especially important when it comes to prescribing medicine. For example, GPs may prescribe more antibiotics because they do not have enough time to educate patients to distinguish between bacterial and viral infections [3]. Additionally, it could be argued that increased workload might lead to higher referral rates as GPs might refer patients to other services to manage their workload. Turner et al. [27] illustrate how increases in emergency department volumes can lead to more referrals to other providers.

The second hypothesis posits that doctors, when experiencing workload pressure, may reduce their reliance on referrals and prescriptions due to difficulties in making sound decisions, encountering patients with unknown medical histories or become less influenced by the expectations of patients [1]. This possibility carries critical implications for patient welfare, as it restricts their access to necessary healthcare services. In this paper, we provide evidence in support of the first hypothesis. The overall results show that GPs increase the intensity of primary care services. They prescribe more drugs, especially antibiotics and they use more assessment referrals. Therefore, doctors are more cautious when they have less time for each patient.

Our results shed light on the risks associated with antibiotic resistance due to a higher rate of antibiotic prescriptions. However, our findings do not suggest any adverse effects in terms of the duration of antibiotic prescriptions. Recent research has shown that shorter antibiotic courses can be equally effective in treating bacterial infections while reducing the risk of antibiotic resistance [25, 30].

We then study whether the change in behaviour is a short-term solution to the daily workload or GPs consider long-term workload when they are under pressure. We investigate the effect of workload on GP doctors on the probability of revisiting the practice for the same condition. Higher workload does not have any significant effect on the probability of revisits. Furthermore, we find that when the workload goes up, patients are moved from in-person to remote consultations and, they are displaced from their named GP to another one. We show that doctors are more cautious in remote consultations which result in more prescriptions. In addition, they try to prescribe more antibiotics when they are not familiar with the patient’s history. It is important to note that our dataset is limited to the pre-Covid-19 pandemic period when the rate of remote consultations was relatively low. The permanent increase in the rate of remote consultations after the pandemic may have a notable effect on referral and prescription decisions of doctors.

Furthermore, we explore the heterogeneity of these effects across different patient groups, revealing that the impact is significantly different for individuals aged 65 or above compared to other age groups. Therefore, elderly patients are the main drivers of our results. Additionally, our analysis considers the heterogeneity among practices, uncovering that the magnitude of the workload effect is more pronounced in short-staffed practices. Practices with lower number of GPs per capita are more vulnerable to the fluctuations in workload. This emphasizes the fact that short term workload fluctuations are echoed in places with long-term excessive workload.

## Literature review

Previous studies have provided insights into the impact of workload on the performance of doctors, particularly in hospital settings. For instance, Batt and Terwiesch [1] examined data from emergency departments in the UK and found that doctors adjust their behaviour during periods of increased crowding by initiating tasks earlier and reducing tasks to expedite service provision. Similarly, Kc and Terwiesch [13] focused on inpatient appointments and demonstrated that a 10% increase in service load led to a reduction of two days in the length of stay for cardio-thoracic surgery patients.

Moreover, behavioural adjustments made by healthcare providers in response to workload have been associated with adverse health outcomes for patients and lower reimbursement for providers. Kim et al. [15] demonstrated that higher intensive care unit (ICU) congestion was linked to a 9% decrease in ICU admissions for eligible patients, subsequently increasing the likelihood of readmission by 32%. In a study by Powell et al. [20], it was observed that when discharging physicians in hospitals were busy, the proportion of patients assigned a “high-severity” status for reimbursement purposes, which corresponded to a 47.8% higher payment for the hospital on average, was significantly reduced.

The available evidence regarding the impact of workload pressure on primary care re-mains limited. This scarcity can be attributed, in part, to the inherent challenges associated with measuring the quality of care within primary care settings.

The existing literature on the impact of workload pressure in primary care settings presents mixed findings. Notably, a study by Shurtz et al. [24] focusing on primary care doctors in middle east demonstrates that increased workload pressure leads to shorter visit duration and a reduction in diagnostic inputs, such as referrals to secondary care and test laboratories. Similarly, research conducted by Freedman et al. [7] in the United States reveals that time-constrained doctors document fewer diagnoses, resulting in an increase in low-value care and a decrease in prescriptions for opioids and preventive care.

In contrast, a study by Neprash [19] suggests that physicians, when faced with workload pressure, intensify their follow-up care for new conditions. This includes an increase in referrals to secondary care and prescriptions for opioids and antibiotics, while decreasing such intensity for established conditions. These varied findings highlight the complexity of the relationship between workload pressure and healthcare outcomes in primary care, emphasizing the need for further investigation and contextual analysis.

Our study stands out from previous research due to the specific context of the 10-minute appointment rule implemented by the Royal College of General Practice in the UK. With appointments already scheduled in tightly constrained time slots, there is limited flexibility to adjust consultation times when the practice becomes crowded. Additionally, the increased workload is encroaching on doctors’ breaks and in-between time, further exacerbating the pressure they face (see e.g., [6, 12, 23]).

Our study's unique dataset allows us to explore nuances like prescribing intensity, GP displacement, and staff substitution, areas less examined in prior work. These insights underscore our contribution to understanding the pressures within primary care under high workload.

## Institutional setting

The primary care sector plays a central role within the UK’s healthcare system, known as the National Health Services (NHS). Despite accounting for only 8% of the NHS budget, primary care is responsible for over 90% of patient contacts [8]. This sector serves two crucial functions: the management and monitoring of long-term conditions, and the control of patient flow to secondary care providers through its gatekeeping role. By fulfilling these roles, primary care effectively alleviates the strain on more costly healthcare sectors, particularly hospitals, thus promoting overall system efficiency and resource allocation.

NHS is mostly tax-funded.^1^ Healthcare services in the UK are provided free of charge at the point of use. It is mandatory for all individuals to register with a general practice contracted with the NHS. General practices are remunerated by the NHS to offer essential primary care services to their registered patients, which involves managing patients with various acute, chronic, and terminal diseases during normal working hours (0800-1830).

In the UK healthcare system, patients are required to obtain a referral from their registered GP in order to access any form of hospital care, even if they choose to receive treatment from a private hospital. As a result, all records of referrals to secondary care are maintained by the patient’s GP. This is the function that is called gatekeeping role of GPs. In addition to managing the access to secondary care services, another important role of GPs is the provision of primary care services through various means including prescribing medications. GPs have the authority to prescribe medications to their patients based on their clinical judgment and the specific needs of each individual.

Despite the pivotal role of the primary care sector, GP doctors are facing mounting pressure attributed to an increased demand for their services and ongoing challenges in recruiting and retaining medical professionals. The 11th National GP Work Life Survey conducted in England revealed that one third of doctors expressed contemplating leaving direct patient care due to the additional workload pressure they face. This worrisome trend suggests that workload pressure has the potential to exacerbate the shortage of GPs by prompting them to exit the profession earlier than anticipated.

Furthermore, the survey highlighted that 60% of doctors reported working beyond their designated working hours, exposing them to a heightened risk of burnout. This concerning statistic underscores the need for urgent attention to address the impact of workload pressure on the wellbeing of healthcare providers. As doctors increasingly voice their concerns, it becomes evident that delays in patient care and the challenge of managing lengthy waiting lists are key issues that need to be effectively addressed within the primary care system.

## Data

The dataset used for this study is sourced from the Clinical Practice Research Datalink (CPRD) Aurum database. CPRD compiles de-identified patient data from 2400 GP practices (approximately 37% of all practices) located throughout England. The data is representative of English population [10, 31]. It’s important to note that, starting in 2018, patients were given the option to withhold their information from being utilized for research purposes, and approximately 2.7% of patients have chosen to exercise this opt-out option [31]. Additionally, the data is enriched with patient-level and practice-level deprivation scores,^2^ providing additional contextual information for analysis.

Our study is based on a random sample of 50 GP practices located in London, collectively catering to approximately 600,000 patients as of 2019. We focused on London to focus on an area where group practices are common which was crucial for our identification strategy. CPRD also has highest coverage in London [31]. The dataset is structured at the level of individual medical observations, captured during patient appointments. Within each observation, we have access to pertinent information such as the staff member’s code and role, as well as the medical code associated with the appointment. Additionally, the dataset encompasses key demographic attributes of patients, including their age, gender, and date of registration at the practice.

Notably, our dataset spans the complete medical history of patients, affording us the ability to extrapolate their underlying health conditions. However, for the specific analysis at hand, we are concentrating on the most recent pre-pandemic period, encompassing the years 2012 through 2019. This temporal focus enables us to rigorously explore the subject matter of interest within a relevant time frame.

Our analysis is specifically centred on appointments conducted by GP doctors. Within this context, we are primarily concerned with gauging the outcomes of these appointments. To this end, we place particular emphasis on the referral and prescription decisions made by the GP during the course of the appointment. These decisions serve as key indicators of the subsequent course of medical care and treatment for the patients involved.

In Panel A of Table 1, we present an overview of essential practice characteristics. Each practice in the dataset comprises approximately 12,000 registered patients and employs a staff of 10 individuals, with 5 of them being GP doctors. On average, each patient participates in around 3.5 consultations per year at the practice, and approximately 2.9 of these consultations involve interactions with doctors. GP doctors, on the other hand, handle an average of 18.5 consultations per day. These statistics provide a snapshot of the patient load, staffing, and consultation dynamics within the examined practices during our study period.

**Table 1:** Summary statistics

	Num. unique obs	Mean	St. Dev
Panel A			
Number of staff per practice per day	80,390	10.08	12.30
Number of GPs per practice per day	97,370	5.25	6.36
Number of registered patients per practice per year	50	11,973.000	13,531.020
Number of total consultations per patient per year	3,442,700	3.49	6.21
Number of GP consultations per patient per year	3,304,261	2.85	4.35
Number of GP consultations per GP per day	511,618	18.46	12.77
Panel B			
Age	598,650	35.903	21.149
Share female	598,650	0.505	0.500
Share in least deprived areas	598,650	0.034	0.182
Share in most deprived areas	598,650	0.082	0.274
Share with at least 1 morbidity	598,650	0.427	0.495
Panel C			
Referral rate (%)	9,445,290	1.71	12.98
Presc. rate (%)	9,445,290	33.99	47.37
Frequent drug presc rate (%)	9,445,290	9.99	29.99
Opioid presc. rate (%)	5,155,865	0.85	9.22
Antibiotic presc. rate (%)	9,445,290	8.08	27.25
Mental health presc. rate (%)	9,445,290	3.59	18.59
QOF incentivised presc. rate (%)	9,445,290	4.13	19.89

In Panel B of Table 1, we present the demographic attributes of the patients. On average, the patients are approximately 36 years old as of 2019. Around 50% of the patient population identifies as female, and a significant proportion of 8.2 % resides in areas characterized as the most deprived. Approximately 42% of patients have been diagnosed with at least one underlying health condition during the period under study. These health conditions are determined using the Cambridge disease code list (for a comprehensive list of conditions and their corresponding prevalence rates, please refer to the appendix). These demographic insights provide a foundation for understanding the patient population within the data set. Moving to Panel C, we focus on summarizing the main outcome variables. Referrals to secondary care are observed in 1.7% of consultations, while prescriptions are noted in 34% of cases. We also show the patterns of prescriptions by some drug category. We follow established literature by selecting drugs that are frequently studied in primary care settings [3, 5, 14].

The first category of drugs we include is frequent drugs.^3^ These include over-the-counter drugs and drugs frequently used to manage cardiovascular disease.

Additionally, we investigate three categories that are of special interest in drug interaction and misuse literature: opioids,^4^ mental health drugs,^5^ and antibiotics.^6^ We also examine drugs for which prescription optimization is incentivized through the Quality and Outcome Framework (QOF), a financial incentive scheme to enhance primary care quality in England.^7^ These QOF incentivized drugs are primarily related to cardiovascular disease management.

When delve deeper into specific drug categories, we find that opioids, antibiotics, mental health drugs, QOF incentivised drugs are prescribed in 0.85%, 8.1%, 3.6%, and 4.1% of all appointments, respectively. These statistics provide an understanding of the prevalence of various medical interventions within the dataset.

## Identification strategy

We aim to estimate the following regression to infer the causal effect of GP workload on the outcome of interest.

1 $$R_{adij} = \alpha_{1} * Num.Appo{\text{int}}_{jd} + \alpha_{2} X_{i} + \gamma_{j} + \tau_{t} + \in_{adij}$$

where *R*_*adij*_ is the outcome. In linear probability models, it is a dummy variable which takes one if the patient *i* in appointment *a* in day *d* by GP *j* is referred or received a prescription. *Num.Appoint*_*jd*_ is a measure of workload which is the number of appointments GP *j* has in day *d*. *X*_*i*_ is a set of patient-level control variables such as gender, age and underlying health conditions and the IMD score of the practice. *γ*_*j*_ is the GP fixed effect,^8^ and *τ*_*t*_ is a set of time dummies including year, month and day of the week fixed effects. We have clustered standard errors at GP level.

**Fig. 2:**
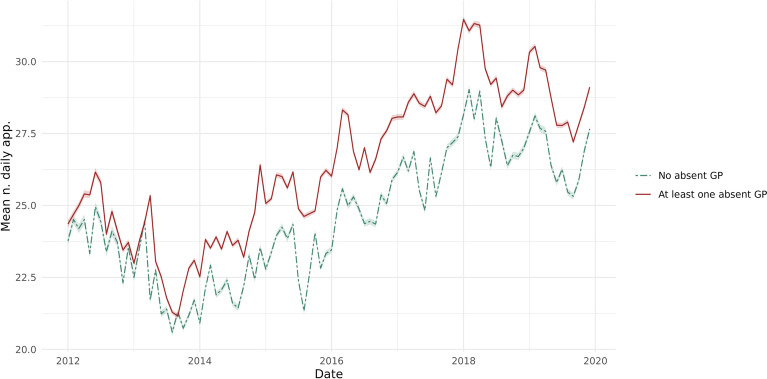
Mean daily appointment per GP by when there is an absent GP and when there is none

There is an endogeneity issue with the above specification, which is due to the sorting of doctors with intrinsic motivation for referral in different types of practices in terms of crowdedness. To overcome this issue, we employ an instrumental variable (IV) approach. We use the temporary absence of a GP colleague as a random shock to the number of present GP’s daily appointments. We measure absence if a GP is present in the practice in the 7 days around day *d* but is not present on day *d*. Figure 2 shows graphic representation of first stage relationship between our IV and the endogenous variable. When a GP is absent in the practice, the workload on present GPs increases.

Table 2 shows the first stage regression. One absent colleague significantly increases the number of daily appointments by 0.36, which is equivalent to a 2% increase. Our IV is strong with a F-statistics above 10 (See Fig. 9 for a reduced form plot of the relationship between the outcome and the IV variable).

**Table 2:** First stage of the 2sls regression

	(1)	(2)	(3)
	Num. appoint	Num. appoint	Num. appoint
Absence	0.365^***^	0.363^***^	0.363^***^
	(0.071)	(0.070)	(0.070)
F-statistics of excluded var	26.14	26.89	26.89
Patient char. Control	No	Yes	Yes
Chronic condition control	No	No	Yes
Time FE	Yes	Yes	Yes
GP FE	Yes	Yes	Yes
Observations	9,445,290	9,445,290	9,445,290

Table shows the first stage regression of the 2SLS model. Num. appoint. represents number of daily appointments per GP. Absence represents number of absent GP per day. We control for the following variables: IMD score ranks patients into 20 categories based on their level deprivation. 1 is assigned to the least deprived patient and 20 to the most deprived one, age and gender of the patient and if they have any chronic condition. Standard errors are clustered at physician level

∗p < 0.1

∗∗p < 0.05

∗∗∗p < 0.01

Please note that by controlling for GP fixed effects and focusing on daily activities, we exclude factors that affect specific practices over the long term. For instance, if a practice experiences a shock to the number of patients, our approach compares only the days when a particular GP is busy with the days when the same GP is less busy, thereby isolating the impact of workload without the influence of long-term practice-level changes. For the factors that influence both GP workload and the outcome in the short term, we argue that our IV satisfies the exclusion restriction assumption, and the absence variable does not directly affect the outcome variable. We consider cases under which this assumption might be violated and explain why we exclude them as serious concerns.

First, there might be some seasonal factors that affect the probability of getting a referral/prescription as well as sick leaves. For example, a flu pandemic might increase both absences and secondary care referrals. We control for various time dummies, such as year, month and day of the week, to reduce the chance of any seasonality in the data. We control for the day of the week to exclude the effect of part time working by some staff and maintain the nature of randomness of our IV.

Second, when a colleague is absent, patients might be directed towards doctors with a higher intrinsic motivation for secondary care referral or prescription. For example, if younger GPs have systematically different referral rates and at the same time are considered to visit extra patients when someone is on leave, this may violate the assumption of the model. However, by controlling for physician fixed effects, we explore within-physician responses to changes in workload. Therefore, we are not concerned about how patients are distributed among doctors when a colleague is absent.

A third concern is that patients with specific characteristics cancel their appointment when their preferred GP is absent. We do not rule out the fact that some patients do not visit the practice when some doctors are absent. That is why the increase in the number of daily appointments in the first-stage regression is small. However, we argue that this cancellation of appointments is not correlated with our outcome variable. First, note that we are considering short-term absences to maintain the feature of randomness in our IV variable. It is not common that a short absence is informed to patients, many of whom have booked the appointment in advance. To reinforce our argument here, in column 3 of Table 2, we show that adding a chronic condition dummy does not change the coefficient on absence, which reassures that patients’ case mix does not affect the correlation between workload and our measure of absence.

In addition, in Table 3, we aggregate the data at the practice level and show that there is no change in the share of patients with underlying health conditions, elderly patients, and those living in the 10% most deprived neighbourhoods when comparing practices with an absence with those with no absent GP. Consequently, we argue that although some patients cancel their appointments in busy days, we rule out the correlation between patient cancellation and patient case mix.

**Table 3:** Exclusion restriction test

	(1) Share Chronic cond	(2) Share elderly	(3) Share deprived
Absence	− 0.0005	− 0.001	0.0003
	(0.001)	(0.001)	(0.0003)
Controls	Yes	Yes	Yes
Observations	94,361	94,361	94,361

Table shows regression of the average characteristics of patient at practice level on the daily number of absent GP. Standard errors are clustered at practice level

∗p < 0.1

∗∗p < 0.05

∗∗∗p < 0.01

## Results

In this section, we first examine the patterns of GP workload and its distribution across various GP roles. We then present our main findings, highlighting how this workload impacts patient outcomes, specifically in terms of referral and prescription patterns. Following this, we analyse the contextual factors influencing these results, including changes in consultation modes and the displacement of patients from their named GPs. Finally, we conduct a heterogeneity analysis, exploring variations in outcomes based on both patient and practice characteristics.

### The burden of workload by staff type

In the first stage regression, we demonstrate that when there is a shortage of GPs, the workload on other GP doctors increases. It’s important to delve into how this extra workload is distributed within the practice among different staff types. The first three columns of Table 4 present similar results to those in Table 2, but they are separated based on the type of GP conducting the consultation. We categorized GPs into three groups: partner GPs who are practice owners and share the responsibility and profit of the practice, salaried GPs who work at the practice based on a contract with partners, and temporary GPs such as locum and registrars. Locum GPs are freelancers, and registrars are junior doctors in the GP training stage. Approximately 77% of GP appointments in our sample are with partner GPs, while the remaining appointments are equally divided between salaried and temporary GPs. In Column 4 of Table 4, we also examine the effect of the absence of a GP doctor on the number of daily appointments by nurses to assess the degree overflow of workload of GPs on other staff types.

**Table 4:** First stage regression by GP type

	Partner GP	Salaried GP	Locum/registrar GP	Nurse
	(1)	(2)	(3)	(4)
Absence	0.439^***^	0.100	0.173^***^	0.042
	(0.095)	(0.066)	(0.041)	(0.052)
Observations	7,396,596	950,903	1,097,791	1,222,663

Table shows the first stage regression of the 2SLS model separately by the staff type of responsible staff. Num. appoint. represents number of daily appointments per GP. Absence represents number of absent GP per day. IMD score ranks patients into 20 categories based on their level deprivation. 1 is assigned to the least deprived patient and 20 to the most deprived one. Standard errors are clustered at physician level

∗p < 0.1

∗∗p < 0.05

∗∗∗p < 0.01

When an absent GP occurs, we do not observe any effect on salaried GPs and nurses. This is intuitive as the workload of these staffs is contracted by the practice. However, the workload on partner GPs and temporary GPs increases significantly. The workload on partners rises by 0.44 appointments, equivalent to a 2.5% increase. This effect is more than twice larger than the results for temporary GPs. Partner GPs hold responsibility for the smooth functioning of the practice, which explains the substantial workload increase during shortages. Workload plays a role in the declining share of GP partners in England. A notable Pulse survey revealed that numerous partner GPs are contemplating the transition to becoming salaried, and many young GPs are not considering partnership at all due to heightened workload pressure [21]. Nonetheless, the role of partners remains pivotal in managing the primary care needs of the local population, as they negotiate contracts with local commissioners and make decisions about the type of services to provide.

Absence of GPs have a minimal effect on the workload of nurses. The coefficient on absence is negligible when considering the number of daily appointments per nurse. There is a substantial body of literature on the advantages of increasing the number of nurses in cases of GP shortages. Many studies have demonstrated positive outcomes in various aspects of care [4, 18, 28]. However, we could not observe this in our analysis. Instead, our study highlights the importance of locum contracts in face of practice volume fluctuations.

### Change in referral and prescription patterns

Table 5 presents the second-stage results of the model for the probability of referral to secondary care (expressed in percentage form) as well as the probability of getting any prescription.

**Table 5:** Second stage regression—referral and prescription probabilities

	Prescription						
	Referred	Any	Frequent	Opioid	Antibiotics	Mental	QOF ind
	(1)	(2)	(3)	(4)	(5)	(6)	(7)
Num. Appoint	0.002	0.052	− 0.026	− 0.008	0.136^∗∗^	− 0.030^∗^	− 0.032
	(0.015)	(0.069)	(0.028)	(0.006)	(0.067)	(0.018)	(0.021)
Mean dep. var	1.71	33.99	9.99	0.85	8.08	3.59	4.13
Controls	Yes	Yes	Yes	Yes	Yes	Yes	Yes
Observations	9,445,290	9,445,290	9,445,290	9,445,290	9,445,290	9,445,290	9,445,290

Table shows the second stage regression of the 2SLS model. All columns are linear probability models where the dependent variable takes 100 if the patient is referred/prescribed drug and 0 otherwise. Num. appoint. represents number of daily appointments per GP. Standard errors are clustered at physicians’ level

∗p < 0

∗∗p < 0.05

∗∗∗p < 0.01

The primary coefficient of interest, represented by the coefficient on “Num. Appoint.” is not statistically significant and negligible in size when the dependent variable is referral probability (see Table 9 for OLS results). However, when conditional on being referred^9^ we examine the change in the composition of referral types, we observe that the share of assessment referrals goes up which shows that physicians are more conservative in diagnosis when under pressure (see table 10).

In terms of prescriptions, we do not observe any significant effect either, however, exploring different categories of drugs, our analysis reveals a significant increase in the probability of prescribing antibiotics by 21.9% from the baseline and a significant decrease in the probability of prescribing mental health drugs by 10.9% from the baseline per one standard deviation increase in the number of daily appointments per GP (13 appointments per day).

To explore the effect of workload on the quantity and duration of drugs, conditional on being prescribed, we conduct a detailed analysis at the individual drug level by disaggregating the data. In Table 11, we present a similar regression to Equation 1, where the dependent variable is either the quantity or duration of prescriptions.

When examining drug quantities, we focus solely on drugs for which the quantity is measured by the number of tablets or capsules. Our analysis reveals that higher workload is associated with a reduction in the quantity of any prescribed drugs. Specifically, it leads to a decrease in the quantity of antibiotics and drugs used to treat mental illnesses. However, in contrast to these findings, the quantity of opioid painkillers increases, although not statistically significant, as a consequence of higher workload. This finding is consistent with the results presented by Neprash [19].

Furthermore, Table 11 extends our analysis to consider the duration of prescriptions in days. Similar to our previous approach, we apply the same regression to investigate the relationship between workload and the duration of prescriptions. Our findings indicate that higher workload is associated with a significant increase in the duration of QOF incentivised drugs.

In summary, our findings reveal that doctors exhibit an inclination to elevate the likelihood of prescribing medications, particularly antibiotics. As a result, a greater number of patients gain access to drug treatments; however, this tendency is offset by a reduction in the average quantity. Conversely, the prescription of mental health drugs experiences a decrease, not only in terms of probability but also in quantity. This signifies a decrease in both the number of individuals receiving these drugs and the extent of their prescriptions.

### Contextual factors of the results

In this subsection, we delve into the drivers of that underlie the results we presented in the preceding section. We examine three scenarios to shed light on the observed patterns: the influence of conservative decisions with future workload in mind, shifts in the mode of consultation, and the displacement of patients from their named GP to a different one. A named GP is the preferred GP of the patient; however, patients can have consultation with any GP in the practice if they want.

In previous section, we showed that GPs are more cautious in using referrals to assess the condition of patients. In addition, they prescribe antibiotics for more patients, increase the duration of prescription of QOF incentivised drugs however reduce the quantity and probability of drugs to treat mental health. All these are consistent with doctors being more cautious when under pressure. They prescribe more of antibiotics and cardiovascular treatment but refrain from psychotropics which may have serious side effects.

It remains uncertain whether this change in prescription patterns is due to doctors opt for more intensive primary care services to alleviate future workload or it is due to poor primary care services which result in need for revisit later. In Table 6, column 1, we observe the probability of patients revisiting for the same condition within 30 days of their initial visit. Notably, heightened workload does not have any significant impact on the likelihood of revisits. Therefore, we do not find any evidence of propagation of current workload to the future.

**Table 6:** Revisit probability and continuity of care

	Repeated consultation	Telephone consultation	Named GP
	(1)	(2)	(3)
Num. appoint	0.191	0.626^∗∗∗^	− 0.230^∗∗∗^
	(0.184)	(0.138)	(0.066)
Mean of dep. variable	40.08	18.69	15.88
Controls	Yes	Yes	Yes
Observations	9,445,290	9,445,290	9,445,290

This table reports the coefficient on Num. appoint. in the second stage regression for prob- ability of repeated consultation, probability of telephone consultation and probability of consultation with usual GP in percentage form. Standard errors are clustered at physician level

∗p < 0.1

∗∗p < 0.05

∗∗∗p < 0.01

We then try to investigate why doctors change the intensity of primary care service. Cadieux et al. [3] shows that physicians tend to prescribe more antibiotics when they have less time to educate their patients. On the other hand, Butler et al. [2] argue that one reason for inappropriate antibiotic prescription is due to physicians trying to maintain their good relationship with their patients.

Therefore, we further see whether doctors altered the nature of their consultations, transitioning from in-person appointments to telephone consultations^10^ which are generally of shorter duration [11]. This shift in consultation mode has the potential to influence the decision of doctors not just because GPs have fewer time to get enough information about patients’ symptoms to do an informed prescription or to educate the patients in order to manage their symptoms with non-pharmacological methods, but since in remote consultations patients are limited in influencing the decision of doctors.

As shown in column 2 of Table 6, the likelihood of a remote consultation experiences a substantial increase in response to higher workload. Consequently, we deduce that one underlying reason driving our results is the change in consultation mode. It is important to note that our observations are confined to the pre-pandemic period, during which remote consultations were not as prevalent. As remote consultations continue to gain traction, it is critical to recognize that doctors’ behaviour and decision-making may undergo modifications when transitioning from face-to-face to remote interactions.

We also explore whether the observed results are primarily influenced by existing patients or by those patients who were originally intended to be seen by the absent GP but were instead displaced to other doctors. The significance of continuity of care has been extensively documented in the literature, emphasizing the positive health outcomes associated with an ongoing relationship between patients and their designated healthcare professionals, extending beyond individual illness episodes [9]. The decision of the GP may change when she encounters a new patient due to a lack of familiarity with the patient’s medical history and needs. Similarly, the decision may be distorted when the appointment involves a familiar patient but the doctor is under workload pressure. While displacement itself is unobservable, we can determine whether a patient was attended to by their named GP or not.

Column 3 of table 6 displays the alteration in the likelihood of having an appointment with the patient’s named GP. On average, there is a reduction of 0.23 percentage points (equivalent to a 1.5% decrease from the baseline) in the probability of patients being able to consult with their named GP. This outcome underscores the impact of patient displacement as a contributing factor to our observed findings.

Consequently, we show that patients are moved to shorter and distant consultations. In addition, they are displaced from their named GPs to new GPs when the practice volume is high. Displaced patients have fewer influence on the doctors as their relationship is not as strong which reduce the intensity of primary care service; however, the doctors tend to

increase the intensity of primary care service as they do not have enough time to educate patients. To see which scenario is stronger, we separately look at the effect of workload pressure on referral and prescription outcomes by consultation type and by named/not- named GP in table 7.

**Table 7:** Second stage regression separately by consultation mode

	Prescription						
	Referred	Any drug	Frequent drugs	Opioid	Antibiotics	Mental	QOF ind
	(1)	(2)	(3)	(4)	(5)	(6)	(7)
In-person consultation							
Num. Appointment	0.010	0.114	0.007	* − *0.008	0.150^∗^	* − *0.021	* − *0.021
	(0.020)	(0.096)	(0.029)	(0.007)	(0.090)	(0.017)	(0.021)
Observations	7,680,263	7,680,263	7,680,263	7,680,263	7,680,263	7,680,263	7,680,263
Remote consultation							
Num. Appointment	* − *0.009	0.207^∗∗∗^	0.004	* − *0.005	0.142^∗∗∗^	* − *0.014	0.002
	(0.007)	(0.072)	(0.026)	(0.009)	(0.038)	(0.018)	(0.017)
Observations	1,765,027	1,765,027	1,765,027	1,765,027	1,765,027	1,765,027	1,765,027
With named GP							
Num. Appointment	* − *0.049	0.519	* − *0.082	* − *0.019	0.545	* − *0.065	* − *0.110
	(0.066)	(0.607)	(0.191)	(0.034)	(0.341)	(0.085)	(0.144)
Observations	1,500,370	1,500,370	1,500,370	1,500,370	1,500,370	1,500,370	1,500,370
Not with named GP							
Num. Appointment	0.009	0.034	* − *0.016	* − *0.006	0.113^∗^	* − *0.024	* − *0.021
	(0.012)	(0.059)	(0.023)	(0.005)	(0.058)	(0.015)	(0.015)
Observations	7,944,920	7,944,920	7,944,920	7,944,920	7,944,920	7,944,920	7,944,920

Table shows the second stage regression of the 2SLS model. All columns are linear probability models where the dependent variable takes 100 if the patient is referred/prescribed drug and 0 otherwise. Num. appoint. represents number of daily appointments per GP. Standard errors are clustered at physician level. Each panel shows the results for a separate sub-sample

∗p < 0.1

∗∗p < 0.05

∗∗∗p < 0.01

It shows that the effect of workload pressure is stronger in remote consultations relative to in-person consultations. It underlines the scenario in which busier doctors have less time to educate patients and hence prescribe more drugs. The coefficient for prescription probability is positive and significant among remote consultations. This again enforce the aforementioned scenario.

On the other hand, the next two panels of table 7 shows that the result for antibiotic prescription is only significant when patient are not seen by their named GPs. This highlight the hypothesis that doctors under pressure may visit patients who are not well known to them. Therefore, we conclude, that the main driver of our results is displacement of patients to remote consultations.

### Heterogeneity

**Fig. 3:**
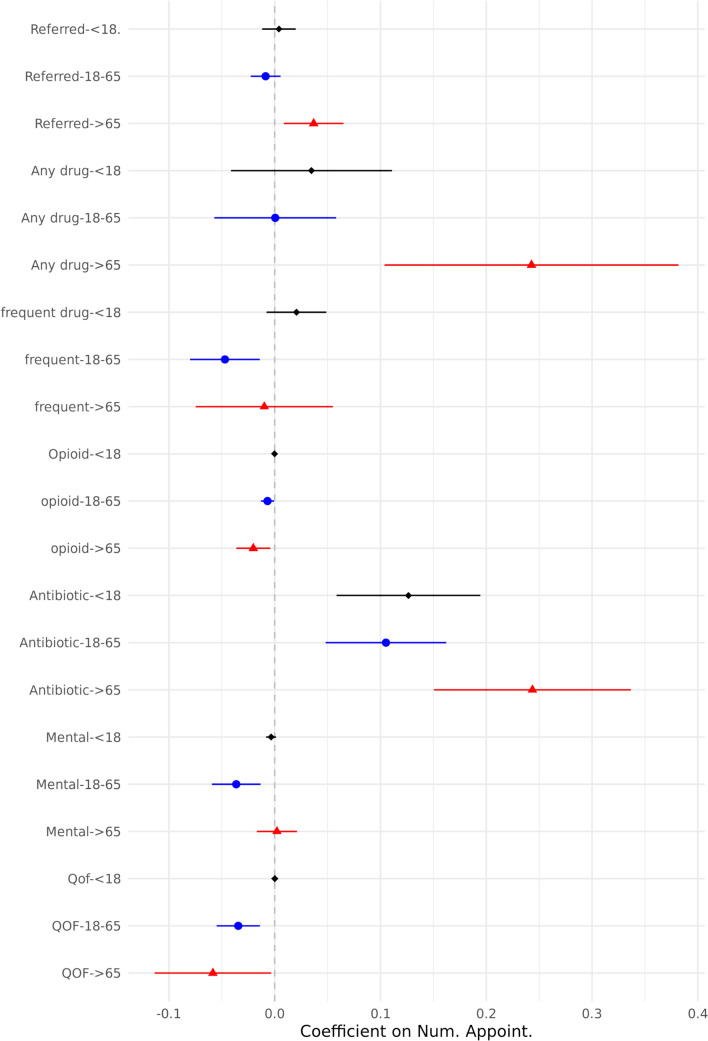
Heterogeneity of outcome variables by Age of patients

In this context, we perform an analysis of heterogeneity based on various patient and practice characteristics. Figure 3 presents the outcomes’ probabilities in relation to patient age. Focusing on referral probability, we find a statistically significant positive coefficient associated with the number of daily appointments among patients aged above 65 years. This is again in line with our previous results that doctors are more cautious when under pressure. Transitioning to prescription patterns, we observe a notable difference in the likelihood of receiving a prescription, particularly antibiotics, between elderly patients and those below 65 years old. In other categories of drugs, the difference between age groups is not statistically significant. Therefore, the response of doctors is mainly in appointments with patients above 65 years of age.

**Fig. 4:**
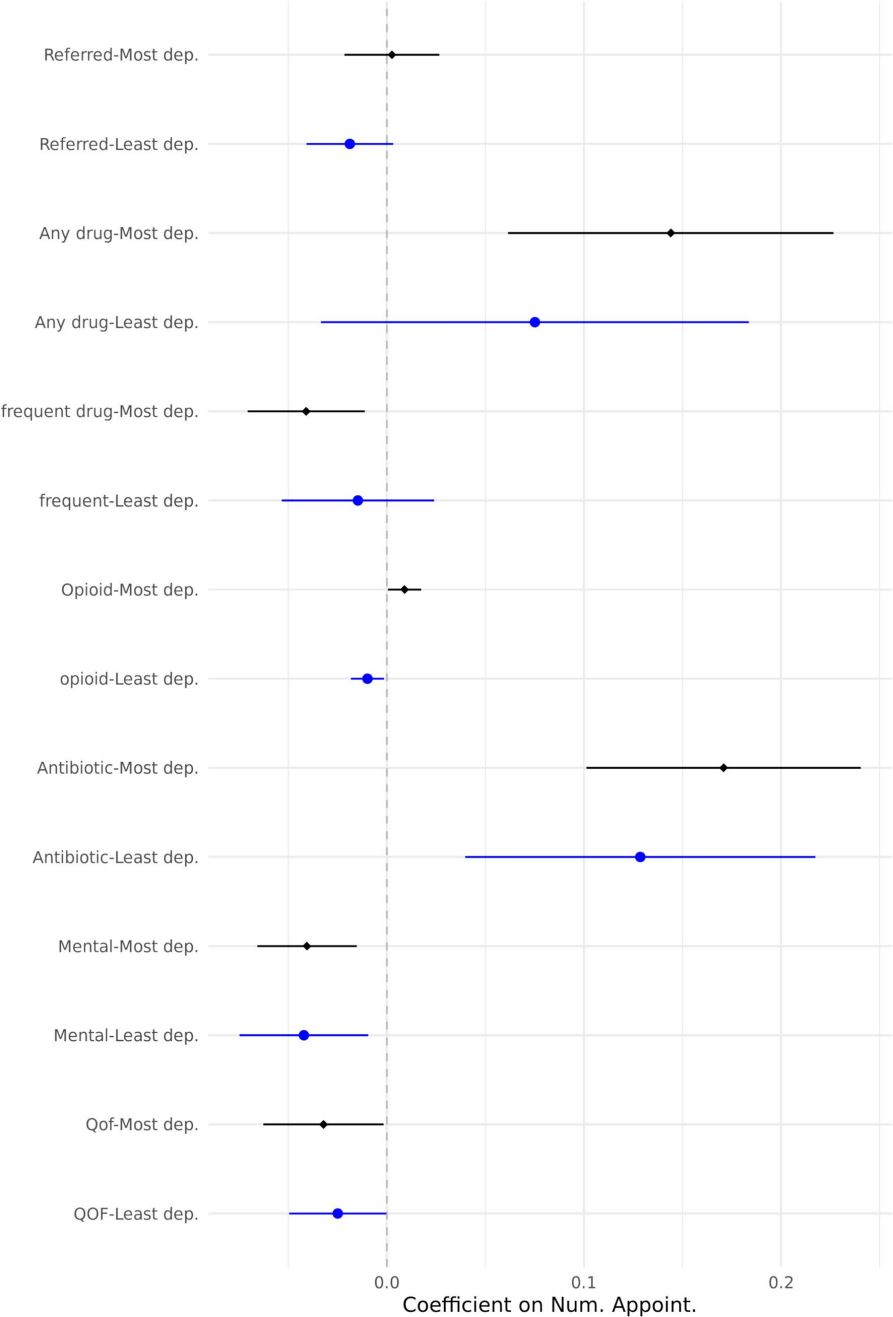
Heterogeneity of outcome variables by IMD score

In Fig. 4, we examine the heterogeneity of these results based on the Index of Multiple Deprivation (IMD) score of patients. We have divided the sample based on the decile of the IMD of patients for those living in 20% most and 20% least deprived neighbourhoods. In relation to referral probability, there is no discernible difference between patients from the most and least deprived areas. In terms of prescription probabilities, we do not see much difference between least and most deprived areas either except for opioid drugs for which we observe completely different patterns among least and most deprived patients. Patients in most deprived areas receive opioids with higher probability while the opposite is true for patients in least deprived areas. Prescribing unnecessary opioids has serious consequences such as adverse effects, habituation, and dependence [22, 26]. Concentration of this response among most deprived patients should be alarming for the health system.

**Fig. 5:**
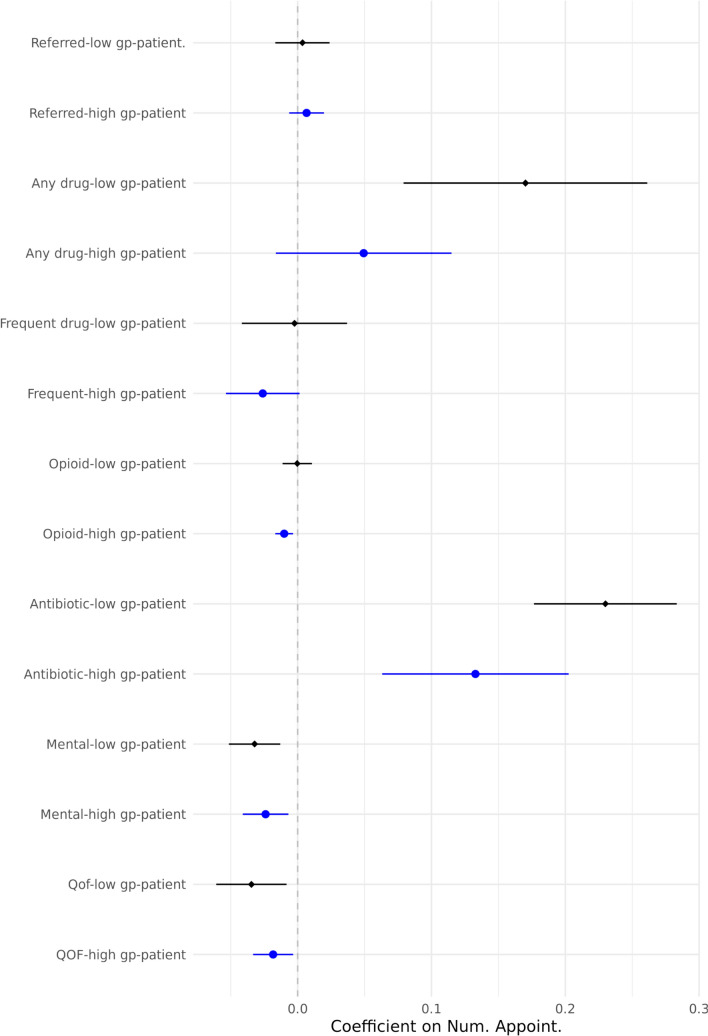
Heterogeneity of outcome variables by per capita GP

**Fig. 6:**
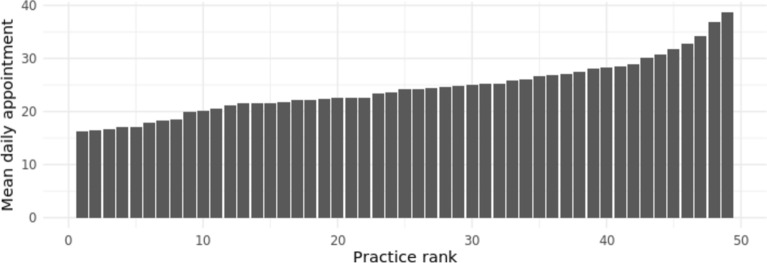
Average number of daily appointments per GP. Note: The figure shows the average number of daily appointments per GP at practice level for 50 practices in our sample. Bars are sorted by the average number of daily appointments per GP

We are interested in examining the differential effects between practices that are already short-staffed and those that are not. Figure 5 presents the results based on the number of GPs per 10,000 patients. Average GP per 10,000 patients is 5.5; we separately look at practices with higher and lower than average GP to patient. We do not observe any noticeable differences in referral probability between the two groups. However, when examining the intensity of prescription, it becomes evident that the increase is predominantly driven by practices which have a permanent GP shortage problem. This finding suggests that the impact of managing workload fluctuations in practices where workload is higher than average is harder and GPs appear to respond more sensitively to changes in their daily workload in these practices.

**Fig. 8:**
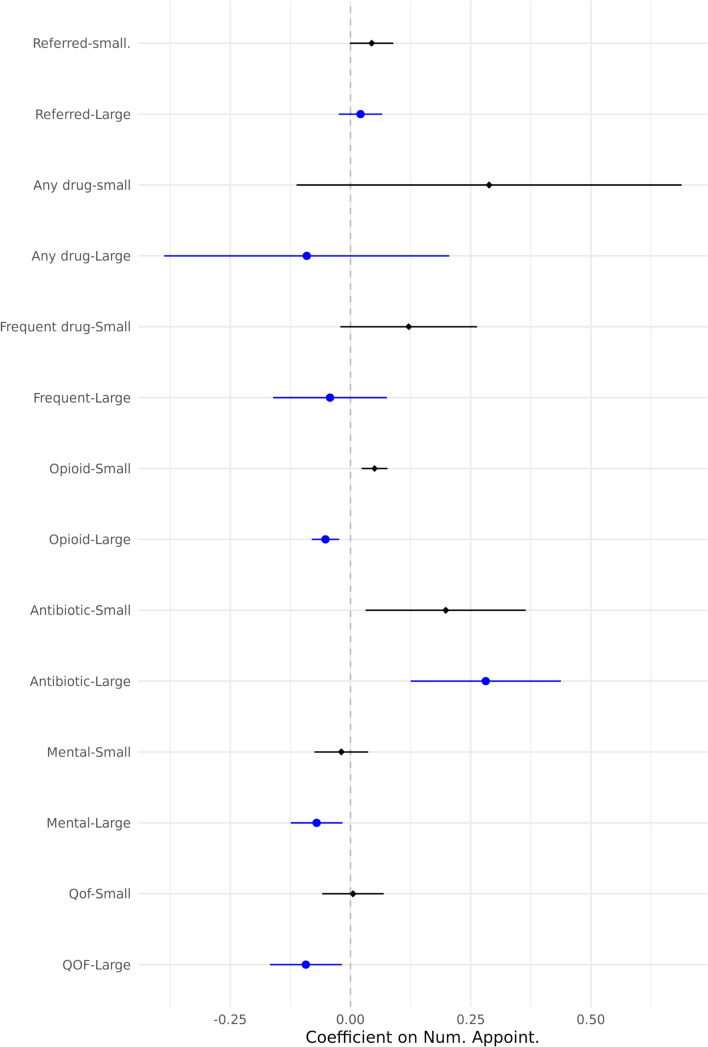
Heterogeneity of outcome variables by practice size of GPs

**Fig. 9:**
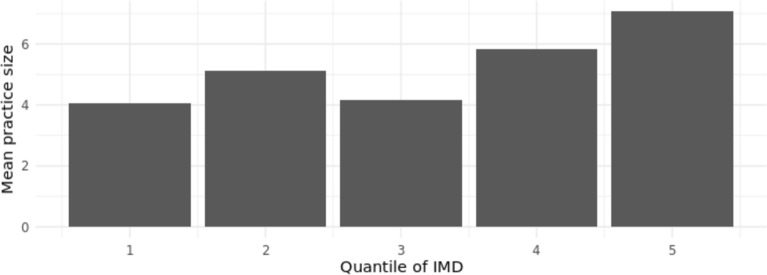
Size of the practice by quintiles of IMD rank. The figure shows the size of the practice in terms of number of GPs (HC) by the quintile of IMD ranking of the practice from 1 (most deprived) to 5 (least deprived)

These figures highlight an issue in the structure of primary care practices in England. Practices in deprived areas are on average smaller in size (they have less than 4 GPs working in the practice, see Fig. 9 and similar results in Fig. 8) and smaller practices have more registered patients per each GP doctor. In addition, they serve more elderly patients. Therefore, our results are driven by specific practices in areas with highest health care need and health inequality. Workload pressure exacerbate this inequality.

## Conclusion

This paper presents empirical evidence demonstrating that an increased GP workload has a significant impact on referral decisions and prescription patterns. Specifically, it finds that higher workload levels are associated with higher share of assessment referrals and an increase in the frequency of prescribing antibiotics. These effects are particularly pronounced among patients aged 65 and above and in practices with limited staffing resources.

Additionally, our analysis reveals that higher workload pressures are associated with a decrease in the quantity of all prescriptions, while simultaneously leading to longer duration of QOF incentivised medications. These findings shed light on the intricate relationship between GP workload and prescription patterns, highlighting potential implications for patient care and health outcomes.

Our study shows that, overall, doctors are more cautious when under pressure and increase the intensity of care. Although patients are not limited in access to health care, inappropriate prescription, especially in the case of antibiotics, can have serious consequences for patients and the society. Therefore, our study shed some light on the public health consequences of workload pressure on doctors.

Furthermore, our study provides valuable insights into the challenges faced by GPs and the potential consequences of high workload levels on referral decisions and prescribing practices. Such findings contribute to a better understanding of the impact of workload pressure on primary care delivery and can inform strategies for optimizing healthcare services and improving patient outcomes.

Our findings have important implications for various aspects of the healthcare system. Notably, they underscore the significance of delivering high-quality care in primary care settings, particularly in systems where primary care doctors play a crucial role as gatekeepers. As the shortage of GPs becomes increasingly concerning in the UK and other regions, our results highlight the potential consequences of higher workload pressures on the existing healthcare workforce.

Our findings highlight the significance of maintaining a balanced staffing approach in primary care settings. In particular, the inclusion of locum and registrar GPs can play a crucial role in mitigating the workload pressures experienced by doctors. The involvement of locum and registrar GPs can provide valuable support by temporarily filling in for absent doctors and distributing the patient load more evenly among the healthcare team. At the same time, the workload pressure is mostly on partner GPs, therefore, encouraging GPs to participate in the practice partnership is equally important in managing workload pressure in primary care practices.

In this research, our identification strategy relies on the use of short-term absence of doctors as an instrumental variable. We utilize short-term absences as they maintain the nature of randomness and are difficult to be substituted, making them exogenous to factors that could affect patients’ outcomes, except for the additional workload pressure on doctors. It is important to note that our findings primarily capture the short-term effects of increased workload. The longer-term implications of workload pressure on doctors, which may have more pronounced effects, are beyond the scope of our study. Additionally, our research does not provide insights into the optimal levels of prescription and referral frequencies. Rather, we demonstrate that increased workload can lead to deviations from doctors’ baseline decision-making patterns.

Due to data access limitations, we could only include information from up to 500,000 patients, equivalent to approximately 50 practices. To avoid potential outliers from practices with unique patient case mixes and workforce structure such as rural practices with a single or few GPs, we focused on practices in London. This decision was made to ensure a sample from a large urban area where group practices are common. However, we acknowledge that our results may not be generalised to rural areas, where workload management dynamics can differ significantly.

It is worth mentioning that due to the unavailability of precise appointment timing data, our analysis focuses on capturing the average effect of workload pressure during a working day. We are unable to specifically examine the impact of fatigue towards the end of the day.

This research opens up several potential avenues for further exploration. One possible direction is to examine non-prescription interventions of GPs another direction is to examine the necessity of referrals to secondary care by analysing conversion rates for specific operations. While our current study cannot distinguish between avoidable and unavoidable referrals and prescriptions, linking primary care data with hospital statistics could provide a means to explore this distinction. By analysing conversion rates, future research could offer a more comprehensive understanding of the referral process and its impact on patient outcomes, highlighting whether referrals and prescriptions are clinically necessary or potentially avoidable.

To enhance the depth of analysis, it would be valuable to incorporate demographic characteristics of GP doctors. For instance, the CPRD gold data includes information on the gender of doctors, which presents an opportunity to investigate potential heterogeneity in workload pressure between female and male physicians. Exploring such variations may provide insights into the differential experiences and responses to workload pressures based on gender, contributing to a more nuanced understanding of the issue.

Expanding the research in these directions would not only enhance the breadth and depth of the findings but also provide valuable insights into the factors influencing referral and prescription patterns and the role of demographic characteristics in shaping workload pressures within the primary care sector.

## Figures and Tables

**Fig. 7:**
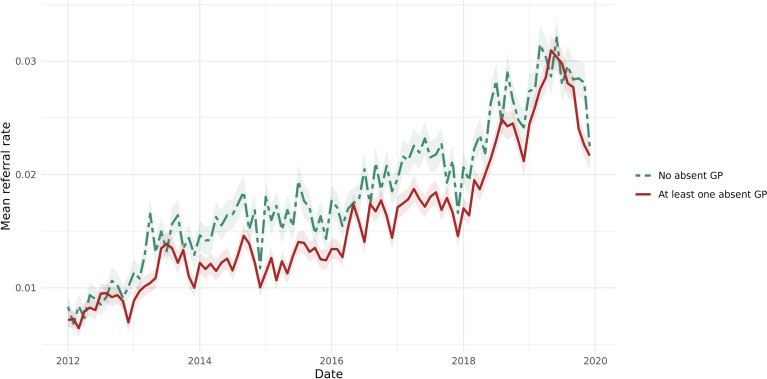
Average referral rate by absence of GPs

## Data Availability

We used deidentified primary care data from the Clinical Practice Research Datalink (CPRD). For more information, please visit: https://www.cprd.com/Data- access, and enquiries can be emailed to enquiries@cprd.gov.uk.

## A. Appendix

See Tables 8, 9, 10, 11 and Figs. 6, 7, 8, 9

**Table 8:** Prevalence of chronic disease

Disease	Prevalence (%)	Num of Obs
Alcohol problems	5.22	28,971
Anorexia or bulimia	0.27	1498
Anxiety & other neurotic disorders	3.66	20,317
Asthma (currently treated)	1.37	7627
Atrial fibrillation	1.44	7975
Blindness and low vision	0.67	3727
Bronchiectasis	0.25	1372
Cancer—[New] Diagnosis in last 5 years	2.08	11,548
Chronic Liver Disease and Viral Hepatitis	1.4	7766
Chronic sinusitis	2.69	14,964
Constipation (Treated)	2.53	14,039
COPD	1.26	7020
Coronary heart disease	2.15	11,911
Dementia	1.05	5844
Depression	7.58	42,097
Diabetes	7.85	43,589
Diverticular disease of intestine	1.6	8904
Epilepsy (currently treated)	0.38	2108
Hearing loss	3.99	22,161
Heart failure	0.74	4124
Hypertension	7.15	39,682
Inflammatory bowel disease	0.56	3126
Irritable bowel syndrome	3.1	17,221
Learning disability	2.46	13,660
Migraine	0.43	2405
Multiple sclerosis	0.15	821
Painful condition	7.35	40,796
Parkinson’s disease	0.14	776
Peptic Ulcer Disease	0.56	3137
Peripheral vascular disease	0.39	2162
Prostate disorders	1.12	6229
Psoriasis or eczema	0.48	2692
Psychoactive substance misuse	1.41	7816
Rheumatoid arthritis, etc	3.26	18,090
Schizophrenia or bipolar disorder	1	5545
Stroke & transient ischaemic attack	1.89	10,512
Thyroid disorders	3.19	17,689
Cancers-other	0.02	99
Diseases of the Circulatory System-other	1.92	10,655
Diseases of the Digestive System-other	6.32	35,082
Diseases of the Ear-other	0.42	2334
Diseases of the Endocrine System-other	1.2	6638
Diseases of the Eye-other	1.84	10,244
Diseases of the Genitourinary System-other	9.34	51,886
Diseases of the Respiratory System-other	1.03	5692
Haematological/Immunological conditions-other	5.62	31,211
Musculoskeletal conditions-other	9.58	53,188
Neurological conditions-other	2.55	14,151
Perinatal conditions-other	0.25	1393
Skin conditions-other	2.87	15,917
No chronic condition	53.91	299,336

**Table 9:** OLS results- referral and prescription probabilities

Prescription							
	Referred	Any	Frequent	Opioid	Antibiotics	Mental	QOF ind
	(1)	(2)	(3)	(4)	(5)	(6)	(7)
Num. Appoint	-0.006^∗∗∗^	0.158^∗∗∗^	0.135^∗∗∗^	0.036^∗∗∗^	-0.003	0.099^∗∗∗^	0.020^∗∗∗^
	(0.002)	(0.012)	(0.011)	(0.003)	(0.006)	(0.007)	(0.007)
Mean dep. var	1.71	33.99	9.99	0.85	8.08	3.59	4.13
Controls	Yes	Yes	Yes	Yes	Yes	Yes	Yes
Observations	9,445,290	9,445,290	9,445,290	9,445,290	9,445,290	9,445,290	9,445,290

Table shows OLS results. All columns are linear probability models where the dependent variable takes 100 if the patient is referred/prescribed drug and 0 otherwise. Num. appoint. represents number of daily appointments per GP. Standard errors are clustered at physicians’ level

∗p<0.1

∗∗p<0.05

∗∗∗p<0.01

**Table 10:** Referral probability by referral type

	Assessment	Treatment	Other	Routine
	(1)	(2)	(3)	(4)
Num. appoint	0.446^∗∗^	0.401	− 0.847^∗∗^	0.176
	(0.207)	(0.437)	(0.383)	(0.214)
Controls	Yes	Yes	Yes	Yes
Observations	161,928	161,928	161,928	161,928

Table shows probability of each type of referral conditional on being referred to secondary care. i.e., the specification is the same as Eq. 1 but the observations are limited to appointments with a referral. Other referrals include referral categories recorded as ‘Unknown’, ‘Self-referral’ and ‘Patient reassurance’. Standard errors are clustered at Physicians’ level

∗p < 0.1

∗∗p < 0.05

∗∗∗p < 0.01

**Table 11:** Prescription patterns

	Num. appoint coef	SD	Obs	Mean of dep variable
Quantity				
Any prescription	* − *1.265^∗∗∗^	(0.229)	2,222,139	101.76
Frequent drugs	* − *0.336	(0.295)	888,364	99.02
Opioid	1.326	(1.130)	70,445	109.66
Antibiotics	* − *0.204^∗∗^	(0.102)	554,818	26.52
Mental	* − *0.225^∗^	(0.128)	328,165	51.43
QOF ind	0.243	(0.272)	381,465	79.57
Duration				
Any prescription	0.043	(0.378)	3,210,498	52.31
Frequent drugs	* − *0.035	(0.234)	943,972	43.60
Opioid	0.076	(0.167)	80,989	22.78
Antibiotics	* − *0.028	(0.070)	763,082	10.47
Mental	0.164	(0.169)	338,786	30.96
QOF ind	0.630^∗^	(0.360)	389,984	50.93

This table reports the coefficient on Num. appoint. in the second stage regression. Panel 1 and 2, conditional on receiving a prescription, shows the quantity and duration of drugs, respectively. Quantity is measured in terms of number of tablets and capsules. Duration is measured in terms of number of days

∗p < 0.1

∗∗p < 0.05

∗∗∗p < 0.01

## Footnotes

1 80% of NHS budget coming from general taxation, 18.5% from National Insurance contributions, and 1.5% from patient charges such as dental fees [16].

2 The Index of Multiple Deprivation is a comprehensive relative measure of deprivation that combines seven domains, including income, employment, education, health, crime, housing, and living environment deprivation. In our context, a practice or patient is considered more deprived if it is in decile 1 or 2 of IMD deciles.

3 We identified the most frequently prescribed drugs in English general practices using the Open-Prescribing project. The ten most prescribed drugs are as follows (BNF chapters in parentheses): Lipid regulating drugs (2.12), Renin-Angiotensin system drugs (2.5.5), Proton Pump inhibitors (1.3.5), Antidiabetic drugs (6.1.2), Selective Serotonin Re-Uptake inhibitors (4.3.3), Beta- Adrenoceptor Blocking drugs (2.4), Non-Opioid Analgesics and Compound Preparations (4.7.1), Antiplatelet drugs (2.9).

4 BNF chapter: 4.7.2.

5 BNF chapters: 4.1, 4.2, 4.3, 4.4, 4.10.

6 BNF chapters: 11.3.1, 13.10.1, 5.1.

7 QOF incentivized drug BNF chapters include: 2.4, 2.5.5, 2.8, 2.9, 2.12.0.

8 We only have de-identified GP id in the data. We cannot observe any characteristic of the doctor other than their role. (e.g., GP partner, salaried GP etc.) in the practice. In terms of the practice, we have only de-identified practice id and can only observe the IMD score of the practice.

9 Repeating the regression of Eq. 1 but limiting the observations to appointments with a referral.

10 They tend to have significantly lower referral rates in remote consultations. On average, telephone consultations exhibit a referral rate of 1%, whereas face-to-face consultations have a referral rate of 2.1%.
